# Lessons Learned during a Rapidly Evolving COVID-19 Pandemic: Aboriginal and Torres Strait Islander-Led Mental Health and Wellbeing Responses Are Key

**DOI:** 10.3390/ijerph20032173

**Published:** 2023-01-25

**Authors:** Patricia Dudgeon, Jemma R. Collova, Kate Derry, Stewart Sutherland

**Affiliations:** 1 Poche Centre for Indigenous Health, School of Indigenous Studies, University of Western Australia, Perth, WA 6009, Australia; 2 College of Health and Medicine, Australian National University, Canberra, ACT 2601, Australia

**Keywords:** COVID-19, Aboriginal and Torres Strait Islander peoples, First Nations, mental health, social and emotional wellbeing, pandemic

## Abstract

As the world journeys towards the endemic phase that follows a pandemic, public health authorities are reviewing the efficacy of COVID-19 pandemic responses. The responses by Aboriginal and Torres Strait Islander communities in Australia have been heralded across the globe as an exemplary demonstration of how self-determination can achieve optimal health outcomes for Indigenous peoples. Despite this success, the impacts of pandemic stressors and public health responses on immediate and long-term mental health and wellbeing require examination. In December 2021, Aboriginal and Torres Strait Islander mental health and wellbeing leaders and allies (N = 50) attended a virtual roundtable to determine the key issues facing Aboriginal and Torres Strait Islander peoples and communities, and the actions required to address these issues. Roundtable attendees critically reviewed how the rapidly evolving pandemic context has impacted Aboriginal and Torres Strait Islander mental health and social and emotional wellbeing (SEWB). This paper presents an overview of this national collaborative consultation process, and a summary of the key issues and actions identified. These results build on evidence from other roundtables held in Australia during 2020, and the emerging consensus across the globe that Indigenous self-determination remains essential to Indigenous SEWB, especially during and following a pandemic.

## 1. Introduction

As the world starts to journey towards the endemic phase following the COVID-19 pandemic, health authorities across the globe are reviewing the efficacy of responses to the pandemic [1]. Aboriginal and Torres Strait Islander public health responses to the COVID-19 pandemic in Australia have been heralded on the global stage as an exemplary demonstration of how self-determination enables optimal health outcomes for Indigenous peoples [2,3,4,5]. Despite this considerable success, there have been concerns regarding the unintended or indirect impacts of public health responses (e.g., lock-downs, and social distancing) and stressors of the pandemic (e.g., uncertainty, loss of income, and housing insecurities) on mental health and social and emotional wellbeing (SEWB), especially for Aboriginal and Torres Strait Islander peoples [6]. Indeed, recent evidence has highlighted some of the negative immediate impacts of the pandemic on mental health for Aboriginal land Torres Strait Islander peoples, including high rates of anxiety and stress during the pandemic [7], increased racial biases [8], and increased suicide rates [9,10]. As the longer-term impacts of the pandemic and pandemic responses start to emerge, it is crucial that the longer-term effects on Aboriginal and Torres Strait Islander mental health and SEWB are reviewed.

In December 2021, Aboriginal and Torres Strait Islander leaders and allies from across Australia came together for a virtual roundtable to determine the key issues facing Aboriginal and Torres Strait Islander mental health and SEWB, and the actions required to address these issues. This paper presents an overview of the collaborative consultation process, and a summary of the key issues and actions outlined in the roundtable final report [11]. The findings within this paper are based on discussions (qualitative data) and reviews that occurred as part of the roundtable meeting, and collectively agreed on by the attendees following the roundtable. These results build on evidence which emerged from earlier public health and mental health roundtables held by Aboriginal and Torres Strait Islander leaders during 2020 [5,12,13], which is that self-determination is essential to the mental health and SEWB of Indigenous peoples.

### 1.1. The COVID-19 Pandemic and Early Public Health Responses in Australia

COVID-19 was first reported in December 2019 and was initially known as the Novel Coronavirus (nCoV-2019). The follow year on 25 January 2020, Australia recorded its first case, and by March 2020, the World Health Organisation announced COVID-19 as pandemic [14]. At these early stages of the pandemic, Aboriginal and Torres Strait Islander leaders and communities were among the first to respond to the immediate public health threat of COVID-19 [1,15,16,17]. By February 2020, Aboriginal Community Controlled Health Organisations (ACCHOs) had already led targeted and effective initiatives to ensure self-determination and preparedness for the pandemic. For example, remote Aboriginal and Torres Strait Islander communities demonstrated self-determination through the closing of their community borders, and the delivery of culturally tailored health messaging in language [18]. The national structure of highly coordinated ACCHOs proved to be an excellent model for driving and supporting public health responses for Aboriginal and Torres Strait Islander peoples across Australia.

Aboriginal and Torres Strait Islander leaders were also driving strategic responses to the pandemic, with wide-reaching public health policy implications. For example, to ensure pandemic preparedness, an Aboriginal and Torres Strait islander COVID-19 Advisory Group was convened between the National Aboriginal Community Controlled Health Organisation (NACCHO) and the Australian Government. This group developed a national pandemic management plan, advising on health issues related to the COVID-19 pandemic response [19]. A largely Aboriginal and Torres Strait Islander academic taskforce also contributed to a chapter within the *Roadmap to Recovery report*, which was presented to the Australian government. Importantly, this chapter outlined four recommendations to ensure the health of Aboriginal and Torres Strait Islander peoples [20]. Importantly, the first of these recommendations emphasised Aboriginal and Torres Strait Islander peoples’ right to self-determination, and the need to empower ACCHOs to lead the pandemic response [13].

The above efforts proved effective in protecting Aboriginal and Torres Strait Islander peoples from exposure, infection, and transmission of COVID-19 in early stages of the pandemic. Historically, Indigenous peoples have been disproportionally negatively impacted by pandemics, due to a range of complex factors (e.g., disproportionate burden of chronic disease, and the social determinants of health) linked to historical and ongoing experiences of colonisation [13]. Despite evidence that pandemics disproportionally negatively impact Indigenous peoples globally [21], Aboriginal and Torres Strait Islander peoples experienced very low rates of infection and fatality during the first two years of the pandemic [4]. In contrast, other Indigenous populations globally were disproportionally negatively impacted by the pandemic [22,23], with fatality rates for some Indigenous communities up to four times that of non-Indigenous peoples [24,25].

### 1.2. The Mental Health Response—The First Roundtable

Recognising that pandemics incur significant mental health challenges and economic burden, a first virtual roundtable was called by the Transforming Indigenous Mental Health and Wellbeing team (https://timhwb.org.au/ accessed on 29 November 2022) at the University of Western Australia (UWA) in April 2020 to explore the mental health and SEWB needs of Aboriginal and Torres Strait Islander peoples as perceived at the early stages of the pandemic. This initial roundtable was made up of 30 Aboriginal and Torres Strait Islander academics, SEWB experts, Elders, health professionals, and representatives from ACCHOs, and non-Indigenous allies. Using a yarning and consensus methodology [26], the group came to a consensus on five recommendations during six weekly virtual roundtable sessions. These recommendations were announced in the National COVID-19 Pandemic Issues Paper on Mental Health and SEWB for Aboriginal and Torres Strait Islander peoples [12]. The recommendations highlighted the importance of (1) self-determination, (2) a culturally safe health and mental health workforce, (3) the social and cultural determinants of health, (4) accessible and culturally safe digital/telehealth, and (5) evaluation which enables Indigenous data sovereignty [5,12].

Although the above recommendations were pivotal in paving a way forward following the immediate threat of COVID-19, they were responsive to the early months of the pandemic in Australia. Since 2020, the pandemic and the public health responses to the pandemic have evolved in unprecedented ways. For example, during 2021, some states (e.g., Victoria and New South Wales) entered strict lockdowns that would last several months. Other states (e.g., Western Australia and South Australia) had virtually no community transmission of COVID-19, and experienced only a few strict snap lockdowns, keeping international and domestic border shut up until March 2022 [27]. There were also new COVID-19 variants, extended lockdowns, vaccine mandates, and constantly changing public health orders and border restrictions. Although these containment efforts were critical for halting the spread of the virus, they unintentionally increased feelings of loneliness [28,29] and also impacted access to Country (the term often used by Aboriginal and Torres Strait Islander peoples to describe the lands, waterways, and seas to which they are connected; this connection is deeply psychological and spiritual and does not translate into non-Indigenous ways of thinking) and cultural practices for some Aboriginal and Torres Strait Islander peoples [30,31]. However, there have also been some unexpected benefits as the result of the pandemic. Some Aboriginal and Torres Strait Islander peoples were able to spend time back in their communities and on Country in order to isolate away from urban centres, there were increased investments in telehealth nationally including the initiation of Aboriginal-specific help-lines, and increased income support payments for low income earners and job seekers [32,33,34]. It is important to reconsider the impacts of the pandemic on Aboriginal and Torres Strait Islander mental health and SEWB in light of this dynamic and evolving context.

Since the first roundtable, there are now emerging empirical data relating to the mental health impacts of the pandemic. Although limited, evidence points towards a negative impact of the pandemic on immediate mental health and wellbeing in the general Australian population. For example, in August 2021, Australia’s national mental health support call centre recorded the busiest days in its 57-year existence [35], pointing towards an increased demand for mental health support services. A report released by the NSW Mental Health Commission also revealed that 60% of NSW residents reported that their mental health was negatively impacted by COVID-19 during 2021 [36].

As a result of the social determinants of health [37,38,39], Aboriginal and Torres Strait Islander mental health and SEWB are more likely to be negatively impacted by the pandemic. Indeed, a report released by the national Australian Mental Health Think Tank in September 2021 highlighted deteriorating mental health as the result of the COVID-19 pandemic, with a disproportionate burden on Aboriginal and Torres Strait Islander peoples [7]. Specifically, the report revealed mental health impacts due to the influence of the pandemic on job insecurity and financial hardships, social networks, and exacerbating existing barriers to accessing mental health treatment. Aboriginal and Torres Strait Islander peoples who were financially unstable experienced greater reductions in wellbeing after the first wave of the pandemic [40]. Rates of suicide also increased by 3.7% for Aboriginal and Torres Strait Islander peoples from 2020 to 2021, while decreasing by 5.4% for non-Indigenous peoples over this same period [9].

There have also been important changes to policy relating to Aboriginal and Torres Strait Islander health and SEWB during the pandemic. Most noticeably, in July 2020, the National Agreement on Closing the Gap was formalised. This landmark National Agreement has been described as marking “a new chapter in the national effort to close the gap between Indigenous and non-Indigenous Australians” ([41] p. 1) and outlines a framework to overcome health inequities experienced by Aboriginal and Torres Strait Islander peoples. The National Agreement is the first to be developed in genuine partnership between the Australian Government and Aboriginal and Torres Strait Islander Peak Organisations (the Coalition of Peaks) and is centred around four priority reforms intended to transform the way that governments work with Aboriginal and Torres Strait Islander peoples. The four priority reforms are (1) the establishment of formal partnerships and shared decision making, (2) building the community-controlled sector, (3) transforming government organisations and (4) shared access to data and information at a regional level [42]. In 2021, the federal government also released the National Aboriginal and Torres Strait Islander Health Plan (2021–2031). This Health Plan [43] was developed in a partnership led by Aboriginal and Torres Strait Islander peoples, reflecting the shift intended by the National Agreement. Together, these policy commitments point towards a positive change in the way that governments work in partnership with Aboriginal and Torres Strait Islander peoples, although the extent to which governments will deliver on these commitments remains to be determined. The context of COVID-19 provided a unique opportunity for governments to progress and deliver on these commitments.

To reconsider the impacts of the pandemic on Aboriginal and Torres Strait Islander mental health and SEWB in this significantly evolved context, a second Aboriginal and Torres Strait Islander roundtable was convened to discuss the ongoing impacts of the pandemic [11]. This roundtable meeting was held in December 2021 and was co-convened by the Transforming Indigenous Mental Health and Wellbeing project at UWA and the ANU School of Medicine, Indigenous Health.

### 1.3. Second Roundtable Context

The first roundtable (April 2020) sessions responded to the immediate threat of the pandemic. In contrast, the second roundtable (December 2021) was convened two years into the pandemic and took a more critical review into how the rapidly evolving pandemic context had impacted mental health and SEWB for Aboriginal and Torres Strait Islander peoples. The second roundtable also provided a timely reflection on the extent to which the Australian Commonwealth Government would implement the recommendations outlined in the first roundtable report, and deliver on other commitments, such as those outlined in the 2020 National Agreement on Closing the Gap [42].

At the time the second roundtable was held (December 2021), there was a national average of about 1500 new COVID-19 cases reported each day, with the majority being from Victoria and New South Wales. This number was growing following the emerging Omicron variant in the Eastern states. In contrast, other states such as Western Australia and the Northern Territory, had no community transmission of COVID-19 and still had stringent border restrictions. There had been just over 2000 deaths from COVID-19, with the majority of these being from Victoria and New South Wales. The vaccination rollout was well underway, with 92.9% of people (aged 16 years and older) having received at least one dose of vaccination. However, only 73.9% of Aboriginal and Torres Strait Islander peoples had received their first dose, with significant variability in vaccination rates between communities and regions.

## 2. Methods

### Collaboration Process

The roundtable was made up of a working party (N = 50) of Aboriginal and Torres Strait Islander mental health experts, community leaders, academics, service providers, representatives from the Aboriginal Community Controlled Sector, and non-Indigenous colleagues. There was also international Indigenous representation from Aotearoa (New Zealand) and Turtle Island (US and Canada). The group participated in the roundtable during a single 3 h meeting which was held online (via Zoom), as detailed further below.

Like the first roundtable, the second roundtable meeting followed a collaborative process aligned with Aboriginal and Torres Strait Islander ways of knowing, being, and doing [44]. As such, the roundtable facilitated open discussions and storytelling relating to the pandemic. Upholding the principle of self-determination, the roundtable was convened and led by Aboriginal and Torres Strait Islander peoples, with majority Aboriginal and Torres Strait Islander representation, and prioritising Indigenous voices.

The roundtable meeting occurred as a semi-structured discussion, led by two Aboriginal facilitators. The meeting began with ceremony: an acknowledgment of Country (due to being on Zoom) from a senior Aboriginal group member, and attendees then introduced themselves, including their name, connection to Country (where relevant), and affiliation. The purpose of these introductions was to build trust amongst the group through connecting participants to each other in a culturally relevant way, and to create a culturally safe space. These introductions are also custom to yarning. Yarning is the way that Aboriginal and Torres Strait Islander people refer to having a conversation. Yarning is a method which privileges Indigenous ontologies and is considered a culturally safe way of engaging with Aboriginal and Torres Strait Islander peoples [26,45].

Following these introductions, international Indigenous representatives from Turtle Island and Aotearoa provided a brief overview of the pandemic in their respective countries. These overviews allowed the group to place the Aboriginal and Torres Strait Islander response to the pandemic within the international context. These overviews were then followed by three brief presentations by Aboriginal members from the group. These members were purposefully selected, as they each lived in different Australian states/territories, and provided different perspectives and experiences of the pandemic. For example, one presenter was an Aboriginal psychologist working in New South Wales, another presenter was an Aboriginal CEO of an ACCHO in the Northern Territory, and the third presenter was an Aboriginal health and SEWB expert working in the Australian Capital Territory. In response to these presentations, the group engaged in unstructured topic yarning altogether, guided by the Aboriginal facilitators. This unstructured yarning did not follow any particular instructions and occurred spontaneously following the presentations.

Topic yarning was then employed by the facilitators to generate more focused and semi-structured discussions around (1) the impact of the pandemic on Aboriginal and Torres Strait Islander wellbeing, mental health, and the social determinants of health, and (2) what is needed to address the biggest uncertainties that communities face in the transition to living with COVID-19. The group was randomly divided into in five small breakout rooms (~8 people in each group) on Zoom to facilitate the yarning. A representative was nominated by each group to share the main messages from their discussion with the wider group. In these discussions, Aboriginal and Torres Strait Islander voices and frontline experiences were prioritised. The facilitators ensured that the discussions remained on-topic, productive, and in line with a strengths-based approach [46,47]. A strength-based approach challenges the dominant deficit narrative surrounding Aboriginal and Torres Strait Islander health and wellbeing, acknowledges the underlying causes of health inequities, and is solutions focused (Forgarty et al., 2018). A team member took notes during the Roundtable discussion, referring back to an audio recording of the discussion to seek clarifications when necessary for the purpose of writing the report.

A smaller working group (N = 4) was convened to synthesise key findings from the Roundtable. This group was comprised of two Aboriginal project leads, who were supported by two non-Indigenous team members from the Transforming Indigenous Mental Health and Wellbeing project. The two Aboriginal members were academics who contributed to the roundtable and provided cultural oversight during the synthesis and writing of the report. One non-Indigenous working group member attended the roundtable to take notes and did not contribute to the roundtable discussions. The other non-Indigenous working group member did not attend the roundtable.

**Figure 1:**
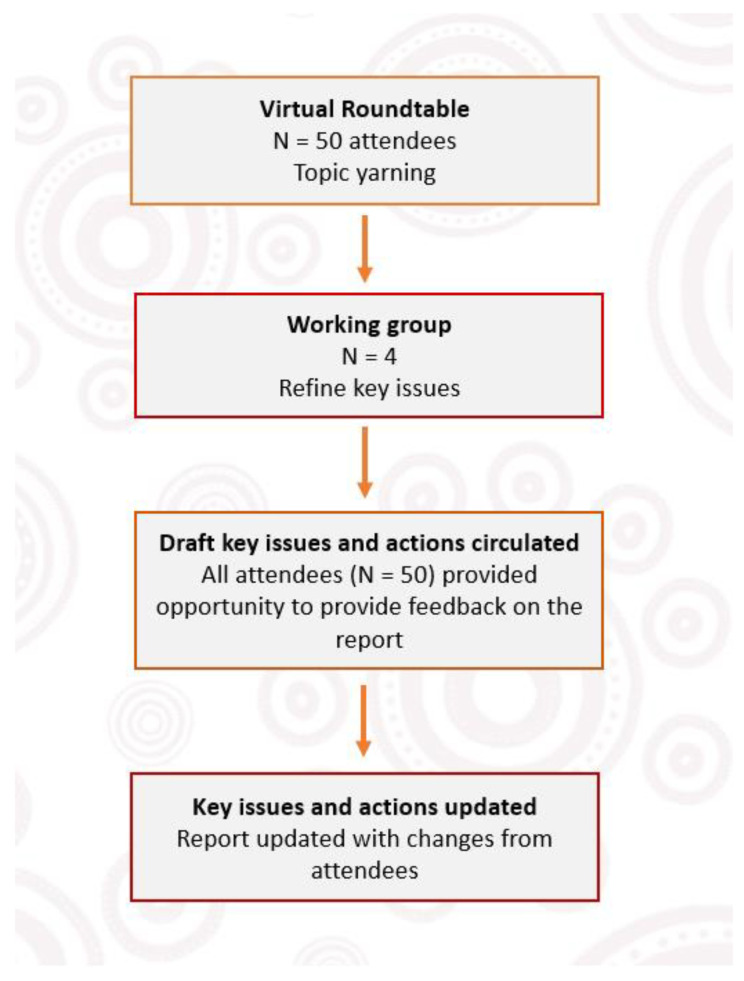
Roundtable Process flow chart.

The working group collaborated online during a 6-month period to develop and refine the key issues discussed during the yarn at the roundtable, and actions required to address these issues (see Figure 1 for process flow chart). This group developed a draft report, which included five key issues which broadly mapped onto the Closing the Gap Priority Reform areas. The report was then circulated to all roundtable attendees via email, who were invited to provide feedback and approval prior to publication. The purpose of this step was to ensure that insights from the yarn were reported correctly and that attendees had an opportunity to provide further input or clarification. Suggestions from the attendees were incorporated into an updated version of the report. People who were invited but were not able to attend the roundtable (including one international First Nations representative from Canada) were also invited to provide written feedback which contributed to the final report [11]. All roundtable attendees were considered authors/contributors on the report as part of a collaborative consultation, as opposed to participants in a research process. Attendees provided verbal and/or email consent to be included as contributors to the report.

## 3. Results: Key Findings from the Roundtable

The stories and views shared during the roundtable revealed that the pandemic had not uniformly impacted all Aboriginal and Torres Strait Islander peoples or communities. Nevertheless, there were common issues shared amongst the group. Below, we detail the key issues identified by the group and the key actions required to address them [11]. For the purpose of this paper, Key Issues 1 and 2 from the Issues paper were synthesised due to the significant overlap in their core issues and actions (Table 1).

### 3.1. A Lack of Cultural Safety in Mainstream Services and Public Health Responses to COVID-19 Has Added to the Cumulative Trauma Experienced by Some Aboriginal and Torres Strait Islander Peoples—Governments Must Uphold Their Commitments to Formal Partnerships and Shared Decision Making to Ensure Cultural Safety

A lack of cultural safety in mainstream services can be a significant barrier to providing appropriate support and deter help seeking [48]. The roundtable group identified that some public health responses to COVID-19 lacked cultural safety and co-design, and contributed to the cumulative trauma experienced by some Aboriginal and Torres Strait Islander peoples, resulting in harm rather than help from these services [11]. For example, many mainstream organisations and public health responses did not consider how historical experiences, such as previous government policies which have harmed and denied autonomy to Aboriginal and Torres Strait Islander peoples, would influence the uptake and acceptance of some pandemic responses and impact wellbeing (for example, increasing feelings of uncertainty, anxiety, and isolation).

Colonisation has left a legacy of trauma, loss and psychological distress for Aboriginal and Torres Strait Islander peoples [49]. This trauma transcends multiple generations, and is still felt today [49,50]. Given this context, some Aboriginal and Torres Strait Islander communities have a justified distrust of information and public health orders imposed by medical institutes and governments [51]. In the context of COVID-19, some imposed public health orders resembled historically traumatic events between Australian governments and Aboriginal and Torres Strait Islander peoples [52,53], and the planning and implementation of these pandemic measures lacked cultural safety, further contributing to cumulative trauma.

The roundtable group identified a key example of a public health response which lacked cultural safety and co-design, and with impacts to mental health and SEWB. Specifically, a lack of cultural safety throughout the vaccine rollout contributed to vaccine hesitancy among some Aboriginal and Torres Strait Islander peoples [11]. Culturally appropriate messaging surrounding the vaccine was developed too late, with frequently changing advice, and lacked a clear voice. Despite early calls of concern regarding the vaccination roll-out from ACCHOs, including the need to engage Aboriginal and Torres Strait Islander peoples from the beginning [54], Western communication styles and language were privileged in government communication strategies. Moreover, there have been reports that racist groups targeted Aboriginal and Torres Strait Islander communities with misinformation to further exploit the vaccine rollout, particularly on social media [55,56]. As a consequence of the above, at the time of the roundtable, vaccination rates were low for Aboriginal and Torres Strait Islander peoples: 54.5% of Aboriginal and Torres Strait Islander peoples had received two vaccinations as compared to 80.6% of the general population [57].

Attendees at the roundtable also shared stories of tension within families and communities regarding whether or not unvaccinated people should be allowed to live with or visit Elders, and how these decisions should be made. The vaccination rollout presented an ethical dilemma, where a justified distrust of the healthcare system was at odds with the actions needed to keep the community safe. Further, the actions required to keep communities physically safe (e.g., lockdowns or restrictions on social gatherings) were sometimes at odds with the actions required to ensure social and emotional wellbeing (e.g., allowing important cultural gatherings and ceremony, such as funerals). What should be the cultural mechanisms of decision making in these complex situations, and who should be responsible for making these decisions?

To address a lack of cultural safety, two key actions were identified in the roundtable report [11]. First, governments must establish formal partnerships and shared decision making with Aboriginal and Torres Strait Islander peoples, including through the co-design of public health responses. This action aligns directly with the first priority reform in the Closing the Gap Agreement [42]. Second, governments must provide or fund services which are culturally safe. ACCHOs are best equipped to respond to the needs of Aboriginal and Torres Strait Islander peoples [58,59], and therefore should be prioritised and funded appropriately (also see Issue 2). In the context of the vaccine rollout, ACCHOs should have been empowered and included to be at the forefront of the national COVID-19 vaccination campaign [60], and funded early to develop and deliver their own place-based messaging. Due to the lack of cultural safety in the vaccine rollout, ACCHOs were left to pick up the pieces, and led a delayed health messaging response following a failed mainstream attempt. Indeed, vaccination rates were high in areas where ACCHOs demonstrated self-determination through leading health messaging and the administration of vaccines [61], such as in Beagle Bay, where more than 90% of the remote Aboriginal community was vaccinated by the end of 2021 [62].

In instances where ACCHOs are unable or not suitable to deliver services, Aboriginal and Torres Strait Islander peoples should be provided with culturally safe services. To ensure cultural safety, all mainstream organisations whose work impacts Aboriginal and Torres Strait Islander people should implement the Cultural Respect Framework [63]. This would include holding all levels of authority accountable to providing culturally safe care, ensuring a culturally safe workforce, engaging in effective partnerships with Aboriginal and Torres Strait Islander peoples, and continuously reviewing cultural safety.

### 3.2. Aboriginal and Torres Strait Islander Mental Health and SEWB Challenges Were Amplified during COVID-19 due to a Lack of Appropriate Consultation with and Resourcing to ACCHOs—ACCHOs Must Be Empowered through Consultation and Needs-Based Funding

There was consensus during the Roundtable that mental health and SEWB challenges were not adequately addressed during the pandemic due to a lack of appropriate consultation with and resourcing to ACCHOs [11]. As mentioned above, ACCHOs have been crucial in leading the response to COVID-19 and taking on extra responsibilities; however, they were not resourced appropriately to deal with these extra demands. For example, the Aboriginal Health Council of Western Australia called for greater inclusion of ACCHOs in the development of government COVID-19 programs, arguing they were not appropriately consulted with [64]. Consequently, ACCHOs raised concerns about this program, including accessibility barriers for Aboriginal and Torres Strait Islander peoples [64]. ACCHOs were required to take on the provision of programs to ensure appropriate support, including providing care packages, food parcels, welfare checks and referrals to SEWB services, but again, were not adequately resourced to do so.

To address the above, the roundtable called for needs-based funding and support for ACCHOs in order to address the disproportionate need and burden of disease experienced by Aboriginal and Torres Strait Islander peoples and communities and for this to be supported by policy that upholds the right to self-determination [65]. This concern is shared by Indigenous peoples across the globe who have long advocated for greater community decision making, consultation and funding for community-driven responses. The Alma-Ata Declaration, a milestone in the field of public health, called for an increase in community participation and focus on social determinants in public health care to address health inequity [66]. Indeed, ACCHOs were heralded at the time as the leading example of how the aims of the Declaration could be operationalised, providing place-based care, and operating within a SEWB framework which acknowledges the social determinants of health.

In the context of COVID-19, the Roadmap to Recovery report [20] estimated that the health needs of Aboriginal and Torres Strait Islander peoples were more than two times that of non-Indigenous peoples, and called for needs-based funding that prioritised ACCHOs. However, despite the Australian Government’s commitment to build the community-controlled sector as part of the Closing the Gap Priority Reforms [42], a continued lack of appropriate funding to ACCHOs and difficulties with commissioning appropriate services that address community needs during the pandemic [67] have been identified as negatively impacting Aboriginal and Torres Strait Islander mental health and SEWB.

### 3.3. COVID-19 Has Exacerbated the Social Determinants of Health and Contributed to Health Inequity—Mental Health Challenges Must Be Addressed through Renewed Policy Focus and Government Funding to Target the Social and Cultural Determinants of Health

The mental health impacts of COVID-19 must be understood in the context of pre-existing social inequities for Aboriginal and Torres Strait Islander peoples. That is, akin to how people with a higher burden of chronic disease (due to the social determinants of health) are more likely to have a severe reaction to a virus infection, people with a high burden of psychological distress and cumulative complex trauma (due to past policies and current social determinants of health) are more likely to be negatively impacted by pandemics [21,68]. A recent report by Australia’s Mental Health Think Tank [7] revealed that the pandemic has exacerbated the disproportionate mental health burden experienced by Aboriginal and Torres Strait Islander peoples. This report specifically highlights the impacts of job insecurity, financial hardships, fragmented social networks, and barriers to accessing mental health treatment, on lower levels of mental health.

The roundtable report [11] specifically highlighted the impact of the COVID-19 pandemic on the social determinants of overcrowded housing, domestic and family violence, food security and access to telehealth (E-mental health). In the context of telehealth, some ACCHOs reported difficulties in uptake due to poor infrastructure, internet and NBN, across both rural and metro regions [69]. A recent review also found that digital technologies can facilitate healing and cultural continuity, but only 63% of Aboriginal and Torres Strait Islander peoples had access to internet at home [70]. These pre-existing inequities limited an effective health and mental health response.

In some cases, public health restrictions put in place to slow the spread of the virus amplified pre-existing disadvantages and inequities. For example, in some communities, families of up to 15 people were reportedly forced to share a single standard household when stay-at-home orders were introduced, with restrictions also placed on their ability to visit Country and engage in wellbeing practices [71,72]. This overcrowding, inappropriate housing, and heightened stress sometimes led to increased spreading of the virus and increases in risk of domestic and family violence [73]. In other cases, public health restrictions limited the protective capability of cultural determinants of health. For example, in the context of food security, some restrictions introduced by local governments prohibited traditional methods of food sourcing and dissemination, which further increased food insecurities for some remote communities [72,74].

To address the above, the roundtable report called for governments to target the social and cultural determinants of health [11]. The social determinants of health have been acknowledged as the root cause of health disparities [38,75]. In the context of COVID-19, the social determinants of health should be addressed through improving household financial resources through increased welfare payments in order to address food and housing insecurities [74], and improving homelessness [76]. Indeed, some government responses to the pandemic did target these social factors, and the expected positive outcomes were evident. For example, in Wilcannia, the New South Wales government leased temporary motorhomes for people to isolate in due to the housing crisis, which enabled the spread of the virus to be controlled [77]. However, discussions during the roundtable revealed fear and uncertainty regarding what would happen when those supports were pulled away.

Aboriginal and Torres Strait Islander peoples have also drawn attention to the importance of cultural determinants of health [78,79,80,81], although these determinants are only starting to be acknowledged by the government. In the context of COVID-19, governments should work with Aboriginal and Torres Strait Islander people and communities to accommodate cultural practices during pandemics. For example, supporting cultural food sourcing during times of lockdown would have helped with food shortages, while also strengthening connections to culture and community, thereby promoting SEWB [78]. Instead, some governments made it very difficult to engage in cultural hunting and gathering practices early in the pandemic by discouraging the sharing of wild meats, as well as through pre-existing stringent licensing legislation [72].

### 3.4. There Are Limited Data Relating to the Impacts of COVID-19 on Aboriginal and Torres Strait Islander Mental Health, and Effective Mental Health Responses—Data Sovereignty and Aboriginal and Torres Strait Islander Governance Are Essential to Building the Evidence Base of What Works for Aboriginal and Torres Strait Islander Peoples

Finally, the roundtable [11] highlighted the need for Aboriginal and Torres Strait Islander peoples and communities to have access to and governance over, timely data relating to the impacts of COVID-19. This key issue clearly overlaps with the fifth recommendation from the first roundtable, which was held in early 2020, and calls for evaluation that upholds Indigenous data sovereignty [5,12]. Priority Reform Four of the National Agreement on Closing the Gap [42] also explicitly advocates for shared access to data and information at a regional level. Without access to accurate, regional data, communities are denied the autonomy to make informed decisions.

Despite the above, data regarding the mental health impacts of the pandemic have been delayed [82]. Often, data fail to record basic demographic information, including whether people identify as Aboriginal and/or Torres Strait Islander. For example, a report on COVID-19 in the Kimberley region (Western Australia’s northernmost region) revealed the absence of basic population data for many communities [17]. Where data regarding the impacts of COVID-19 do exist (e.g., suicide rates for Aboriginal and Torres Strait Islander peoples), they are typically delayed, deficit-focused, and based on Western perspectives of wellbeing.

Although there have been many stories of success for Aboriginal and Torres Strait Islander peoples during the pandemic [4,15,61], there remain limited evaluations of solutions that have been effective in addressing the specific mental health impacts of the pandemic for Aboriginal and Torres Strait Islander peoples. Governments have not adequately engaged with Aboriginal and Torres Strait Islander peoples in appropriate Indigenous evaluation methods and frameworks. Indigenous evaluation methods include evaluating constructs that are meaningful to Aboriginal and Torres Strait Islander peoples, for example, evaluating impacts of the pandemic to connections to Country and community, rather than narrowly measuring changes in suicide rates.

The roundtable report calls for Indigenous data sovereignty as part of a global movement regarding the human right of Indigenous peoples to govern the creation, collection, ownership and application of their data [83,84]. These issues also clearly point to the need to implement the Indigenous Evaluation Strategy proposed by the Australian Government Productivity Commission [85]. This would involve centring Aboriginal and Torres Strait Islander peoples, perspectives, priorities, and knowledges across all stages of evaluation. Future research should build the evidence base of what works for mental health and SEWB of Aboriginal and Torres Strait Islander peoples through celebrating and learning from success stories and taking a strengths- and SEWB-based approach [46].

## 4. Conclusions

In Australia, Aboriginal and Torres Strait Islander-led initiatives have enabled an effective public health response to the COVID-19 pandemic. Despite this success during the first waves, it is imperative that governments also consider the longer-term impacts of the pandemic on mental health and SEWB that will be disproportionately felt by certain populations due to new and pre-existing inequities within the social determinants of health. Aboriginal and Torres Strait Islander peoples are one population that is vulnerable to the health and mental health impacts of pandemics for these reasons.

This paper builds on the Close the Gap Priority Reforms [42] and recommendations from earlier roundtables [5,13], through outlining four key actions to ensure the SEWB of Aboriginal and Torres Strait Islander peoples during and following the pandemic. These actions call for local- and system-level change, including the prioritisation and adequate funding of ACCHOs in order to enable empowerment and self-determination, as well as strengths-based and culturally informed approaches driven by communities, which target the social and cultural determinants of SEWB. These actions also need to include system changes to empower effective responses in the future regarding COVID-19, as well as for any other pandemic.

The results in this paper are specific to Aboriginal and Torres Strait Islander peoples. There is great diversity amongst Indigenous populations globally, and it would be important to consider how the pandemic has impacted the wellbeing of other Indigenous peoples, and any effective strategies which have mitigated these impacts. Our roundtable did have international Indigenous representation from Aotearoa (New Zealand) and Turtle Island (US and Canada), who further reinforced the importance of self-determination.

This paper highlights the continuing need for governments to focus on addressing the four Priority Reforms as outlined in the National Agreement on Closing the Gap [42]. These include (1) formal partnerships and shared decision making, (2) building the community-controlled sector, (3) transforming government organisations, and (4) shared access to data and information at a regional level. These are existing issues which must be addressed to ensure the overall wellbeing and long-term recovery from the pandemic for all peoples.

## Figures and Tables

**Table 1:** Summary of the key issues and actions identified.

Key Issue Summary	Key Action Summary
A lack of cultural safety in mainstream services and public health responses to COVID-19 has added to the cumulative trauma experienced by some Aboriginal and Torres Strait Islander peoples. For example, a lack of cultural safety throughout the vaccine rollout contributed to vaccine hesitancy, with impacts to mental health.	Governments must uphold their commitments to formal partnerships and shared decision making to ensure cultural safety. All mainstream organisations whose work impacts Aboriginal and Torres Strait Islander people should implement the Cultural Respect Framework.
Aboriginal and Torres Strait Islander mental health and SEWB challenges were amplified during COVID-19 due to a lack of appropriate consultation with and resourcing to ACCHOs.	ACCHOs must be empowered through consultation and needs-based funding.
COVID-19 has exacerbated the social determinants of health and contributed to health inequity.	Mental health challenges must be addressed through renewed policy focus and government funding to target the social and cultural determinants of health.
There are limited data relating to the impacts of COVID-19 on Aboriginal and Torres Strait Islander mental health, and effective mental health responses.	Data sovereignty and Aboriginal and Torres Strait Islander governance are essential to building the evidence base of what works for Aboriginal and Torres Strait Islander peoples.

## Data Availability

Not applicable.
